# Values and Behavior Among Minorities in Southwest China: A Cross-Cultural Validation of the Refined Value Theory

**DOI:** 10.3389/fpsyg.2019.01750

**Published:** 2019-08-02

**Authors:** Yanli Yang, Jia Zhou, Pan Feng, Guangyu Jiang, Yan Long, Yong Zheng

**Affiliations:** ^1^Center for Studies of Education and Psychology of Minorities in Southwest China, Southwest University, Chongqing, China; ^2^Teaching Department of Public Course, Ningxia Art Vocational College, Yinchuan, China; ^3^School of Humanities and Management, Southwest Medical University, Luzhou, China; ^4^Faculty of Psychology, Southwest University, Chongqing, China

**Keywords:** southwest Chinese minority, refined value theory, value-behavior relations, Chinese culture, moderating role

## Abstract

The minorities in Southwest China are characterized by a blend of diverse cultures. It is not clear how values predict behaviors in such a population. We applied Schwartz's refined value theory to assess the association between values and behaviors. Respondents (*N* = 532) reported values using the Portrait Values Questionnaire and rated their postulated behaviors with the Everyday Behavior Questionnaire. A confirmatory factor analysis validated the discriminant validity of 19 refined values. Multidimensional scaling analyses revealed a circular motivational structure with national characteristics. More importantly, the correlation analysis showed that 13 of 19 values correlated most strongly with its postulated or adjacent behaviors, which supported the cross-cultural prediction of behaviors by values. Variations differing from theoretical structure and the moderating of value-behavior relations by gender better reflected the effects of Chinese traditional culture, such as harmony, benevolence, and “the Golden Mean.” The results of this study enrich the cross-cultural validity of the refined value theory among Chinese minorities and enhanced our understanding of the multiculturally influenced minority population.

## Introduction

Associations of values with behaviors have frequently been the focus of attention in psychological studies. Many researchers have studied values on the assumption that they can explain, influence, and even predict behavior in hypothetical (e.g., Verplanken and Holland, 2002; Sagiv et al., 2011) or real-life situations (e.g., choosing university courses, voting for political parties; Feather, 1988; Schwartz, 1996). However, similar tests may manifest diverse results in different cultural contexts, which has sparked extensive discussions that took place across cultures on the congruence or divergence of general values and value-behavior relations. Schwartz (2006) noted that the prevailing value emphases in a group may be the presentation of culture of the group, and they convey shared conceptions of what is desirable and important in the culture, and shape and explain the beliefs, actions, and goals of group members. Schwartz and Bardi (2001) reported a largely shared, pan-cultural value hierarchy across more than 55 nations; while a Canadian survey stated that most Ontarians felt that Muslim immigrants had fundamentally different values from them (Keung, 2016). Schwartz et al. (2017) provided more consistent evidence of the values by relating them to behavior in four culturally diverse countries, and Hanel et al. (2018) revealed that people in different countries could differ in behaviors that were regarded as typical as instantiations of values, while nonetheless holding similar views about the abstract meaning, and importance of such values. These results were conducted with people living in a single cultural context; how, then, would a population in a multicultural context appear? In this way, the present study traced this train of thought by exploring the universal value types and the relations of value with value-expressive behaviors in a multicultural population in China.

### The Refined Value Theory

In order to establish the research related to values, it is necessary first to actually identify how many basic human values exist across cultures. Schwartz (1992) defined 10 broad basic values. He later partitioned them into a set of 19 narrower, meaningful, and conceptually distinct values (Schwartz et al., 2012). They are ordered relative to one another on a circular motivational continuum, depending on the compatibility, or conflict between the motivations they express. A value is compatible with others if actions that promote or express it also promote or express the other values. The more closely any two values are located on the circle, the more compatible are their underlying motivations. A value conflicts with others if actions that express or promote it do so at the expense of other values. The more distant any values are on the circle, the more conflicting their underlying motivations. The 19 values can be combined into four higher-order values on two bipolar dimensions: openness to change vs. conservation and self-enhancement vs. self-transcendence. As one of the most mature and widely accepted theories in the field of value research, the refined value model has been assessed in over 19 samples of different countries. Its trans-situational context attracted researchers to apply the model to interdisciplinary and cross-cultural research.

### Associations Between Values and Behaviors

Researchers in this tradition argue that values are important in guiding human behaviors. As “value” is understood as an abstract and vague conceptualization, people usually link values to the concrete actions. Earlier researchers suggested that some values may predict single behaviors (e.g., consumer choices, environmental behavior; Schultz and Zelezny, 1998; Doran, 2009), or that one value may be reflected by sets of behaviors (Bond and Chi, 1997). Later, some researchers studied the strength of association between values and their corresponding behaviors. Bardi and Schwartz (2003) firstly revealed relationships of a comprehensive set of values with a wide range of behaviors using the Schwartz Value Survey (Schwartz, 1992); they found differences among 10 pairs of value-behavior relationships, including that tradition related strongly to value-expressive behaviors but security related marginally to its corresponding behaviors. Schwartz and Butenko (2014); Schwartz et al. (2017) related the 19 narrowly defined values systematically to behaviors, and examined how value tradeoffs predict behavior. Maio (2010) also reported that the same values may be expressed by different behaviors in different social groups, and that the value may not predict atypical behaviors due its lack of association with them. For instance, Turkish people attach the same importance to equality as people in other countries do, but less to gender equality (Hanel et al., 2017). Furthermore, Peng et al. (1997) showed that the measures that identified a behavioral scenario relevant to values yielded reasonable criterion validity in cross-cultural value comparisons, because this may avoid cultural differences in the meaning of particular value terms. Accordingly, culture may be an indispensable factor for value-behavior relations.

### Influence Factors on Value-Behavior Relation

Of many influential factors of value-behavior relations, external pressure is but one. Bardi and Schwartz (2003) claimed that the less external pressure to perform value-expressive behaviors, the stronger is the relation of the value to the behavior. In other words, situational pressure might make group members adhere their behaviors to group expectations rather than based on their own values, which might reduce the relationship of the value with the behavior. This finding achieved wide recognition and was cited in many relevant studies (e.g., Hitlin and Piliavin, 2004; Lönnqvist et al., 2006), but few studies tested it for repeatability. Schwartz et al. (2017) tested the claim in four countries, but found that it was supported only in Italy. Moreover, the claim failed to be replicated in research by Schwartz and Butenko (2014).

“Gender roles have an effect because they convey the costs and benefits of behaviors for men and women,” found Eagly et al. (2004). Thus, gender may be another potential moderator of value-behavior relation. Some cross-cultural evidence shows that gender differences in values and behaviors are linked to more benevolence for women and to more power for men (e.g., Best and Thomas, 2004; Eagly et al., 2004). However, Roccas et al. (2002) combined gender roles with self-conscious deliberation to conclude the strength of the association between value and behaviors, and that people had more self-conscious deliberation before engaging in role-*inappropriate* behavior. If so, the relation between value and behavior should be stronger for benevolence for men and power for women. Although the sources of sex differences in the value-behavior interrelations were explained by evolutionary psychology focusing on humankind's past adaptations (behaviors) (Geary, 1998) and the social role theory focusing on division of labor (behaviors) (Eagly et al., 2000), the growing independence, and equality of women in the diverse social contexts may lead to the changes in cognitive and adaptation mechanisms. These changes may apply to values that were generally viewed to guide behavior. In addition, some studies have reported that gender differences are less consistent across cultures in various value domains (e.g., Struch et al., 2002; Schwartz and Butenko, 2014).

### Overview of the Present Research

We carried out our studies in a multicultural but small population, which has been seldom included in previous publications. The minorities were from Southwest China, mainly including the province of Yunnan, Guizhou, Sichuan, and Chongqing, and comprised more than 30 of the 55 official Chinese minority groups. Each group either inhabits a specific region in Southwest China, or is dispersed among other ethnicities. Despite such high centralized and multicultural minority distribution, Southwest minorities manifested more harmony, and intercultural fusion (Zheng, 2007). Such a social context of multicultural interactions is different from that of a single-minority group living among the ethnic Han. Furthermore, due to geographical remoteness and relatively closed natural environment, exchanges between Southwest minorities rarely occurred outside the Southwest; Southwest China thus presents an unbalanced and multilevel regional development. As recently as the 1950s, the less populous minorities still retained the imprint of ancient tribes, such as the Durong, Nu, and Jinuo (Ren, 2012). In addition, the Southwest minorities have been influenced by diverse cultures of neighboring minority groups, traditional Chinese Confucianism, and modern civilization (Li and Wu, 2017); as members of the Chinese nation, they are also exposed to the national values, which are the common and important criteria to guide actions of the 56 Chinese ethnicities. As a result of this diversity, many different values coexist within the same multicultural space, leading to the potential for “cultural conflict” (Zheng, 2007; Li and Wu, 2017). Such diverse cultural effects make the groups multicultural minorities in China, but the multicultural features are not observed among ethnic Han (see Attachment for a more detailed overview on ethnic minorities in China).

In this study, we followed the same procedure and analyses conducted by Schwartz and Butenko (2014) to validate the applicability of the circular motivational model of the 19 values among the minorities of Southwest China, and to test the associations of values with behaviors and the moderating roles of normative pressure and gender. We expected that the 19 refined values or the 10 original values form a motivational continuum among the minorities in Southwest China. Given the differences in diverse indigenous cultures, our circular motivational structure may be somewhat different from the theorized one; the specific values which constitute the four higher-order types on two bipolar dimensions would be different from the theorized values. Moreover, we expected that each of the 19 values in our population would be more positively related to its postulated behaviors than the other 18 sets of behaviors, and the higher-order values in Southwest minorities would be correlated negatively with inhibiting behaviors that are primarily propelled by motivationally conflict values. Finally, we anticipated that the external pressure and gender would play moderating roles in the relationships of values with behaviors for the Southwest minorities.

## Materials and Methods

### Participants

The final analysis was conducted on 532 surveys in Chongqing, Yunnan, Guizhou, and Sichuan provinces in China. Respondents included members from 31 of the 55 official ethnic minorities in China including Yi, Miao, Bai, Tujia, Hui, Tung, Zhuang, Hani, Dai, Bouyei, Gelo, Nu, Lisu, Jingpo, Shui, Naxi, Tibetan, Wa, Achang, Blang, Jinuo, Qiang, Yao, Lahu, Dongxiang, Pumi, Drung, Manchu, She, Li, and Maonan. Occupationally, 73.9% (*n* = 393) were students, and the other 26.1% (*n* = 139) included teachers, workers, peasants, freelancers, physicians and nurses. In the sample, 53.6% (*n* = 285) were women, 71.4% (*n* = 379) were aged 17–24 years, 18.4% (*n* = 98) were aged 25–35 years, 9.8% (*n* = 52) were aged 36–49 years, 91.9% (*n* = 489) were undergraduates or with higher educational levels, 91.4% (*n* = 486) were non-religious, 81.8% (*n* = 435) were unmarried, 83.8% (*n* = 83.8) were skilled Mandarin masters, 62.4% (*n* = 332) were descendants of the same ethnic minority group, and 69.7% (*n* = 371) were from the minority-inhabited area.

### Procedures

We recruited several minority postgraduates as assistants, who were from the minority-inhabited areas and familiar with their own ethnic group's language and customs. They were trained regarding instructions and testing procedures. During the 7-day holiday of the National Day, the assistants took our paper-and-pencil surveys home to their families (parents, siblings, or relatives) or to their minority acquaintances (e.g., friends and classmates) to fill out. Additionally, two teachers from Southwest Minzu University delivered our questionnaires to their minority students during a class session. The completed questionnaires were collected on the spot within about 30–45 min. The participants' ethnic minority identification was self-reported, and we confirmed their identification through telephone fellow-up. The participants got a monetary reward (10 Yuan RMB) after completing two self-report questionnaires as a token of our gratitude for their participation.

### Measurements

#### Values

We assessed values with a Chinese Simplified Female and Male version of the 57-item Portrait Values Questionnaire (PVQ; Schwartz et al., 2012) separate sex-matched value surveys that differed only in the use of pronouns. Each item has a verbal portrait of diverse people, each implying a value that is held by the person. Respondents' own values were derived from their reports of how similar the person is to himself (herself) in each portrait on a 6-point scale from 1 (*not like me at all*) to 6 (*very much like me*). For example, “Having ambitions in life is important to him” describes a person who emphasizes achievement values; “Being wealthy is important to him” describes a person who values power highly; “Obeying all the laws is important to him” describes a person who thinks that humility is important. The 57 items could be combined into 10 basic values or 19 narrowly refined values. The internal reliability of the 10 values subscales ranged from 0.631 to 0.825 and the 19 values subscales ranged from 0.606 to 0.816 in this study.”

#### Behaviors

We adopted the English version of Everyday Behavior Questionnaire (EBQ; Schwartz and Butenko, 2014). Based on the act-frequency approach, the survey consists of 19 sets of three to six behavioral tendencies, 85 items in total. The behaviors in this measure were generated specifically to match the value dimensions. Respondents were asked to report how often they had engaged in each behavior during the past year on a 5-point scale from 0 (*never did*) to 4 (*always did*). The behavior items match their corresponding values. For example, “Keep promises I made to friends or family” matches benevolence-dependability value; “Learn something simply for the joy of learning” matches self-direction-thought value; “Look for exciting activities to break up my routine” matches stimulation value.

Considering the lifestyle differences, we changed the description of one behavior item, “do things that provide sensual pleasure (e.g., bubble bath, massage)” to “do things that provide sensual pleasure (e.g., shower, enjoying music).” Additionally, respondents needed to distinguish between never having even one opportunity to do so (X) and never performing a behavior despite having had at least one opportunity (0). The response “X” was treated as missing data, consisting of <1% in this study. We employed two independent back-translation procedures to translate the EBQ from English to Chinese. Prior to analysis, we replaced missing data with series mean. The internal reliability of the 19 behaviors subscales in this study ranged from 0.585 to 0.793.

## Results

### Reliability and Discriminant Validity of the 19 Values and 19 Behavior Variables Using Confirmatory Factor Analysis

We used confirmatory factor analysis (CFA) to assess the discriminant validity of the 19 values and behaviors among Southwest minorities. It is impossible to include all 19 latent variables, 57 value items, or 85 behavior items in a CFA model of the whole circle (Harrington, 2008), so we performed four higher-order CFAs (including openness to change, conservation, self-enhancement, and self-transcendence) separately for values and behaviors using AMOS 23.0 (IBM Corporation, Armonk, NY) with maximum likelihood estimator (Schwartz and Butenko, 2014). We used three indexes to evaluate the goodness of fit of the models: the root mean square error of approximation (RMSEA), the comparative fit index (CFI), and the standardized root mean square residual (SRMR). We set CFI values ≥ 0.90 (Bentler, 1990), RMSEA values ≤ 0.08 (Browne and Cudek, 1993), and SRMR values ≤ 0.06 (Hu and Bentler, 1999). We also based the modified indexes on fixing the variance of the latent factors to 1 to achieve identification, estimating the loadings freely, but allowing no covariances between uniqueness (Schwartz, 2014).

#### Values

Seven items with an asterisk in Table 1 were dropped from the original 57 items. Because of the higher cross-loadings of the second item of face value factor (FAC2) on achievement, we shifted it from the former to the latter. FAC2 was relabeled as AC4. We also incorporated three values (humility, hedonism, and face) respectively into higher-order conservation type, higher-order openness type, and higher-order self-enhancement type (Schwartz and Butenko, 2014). The top panel in Table 3 reports the fit statistics for each set of revised values in the four higher-order models, without correlated errors and cross-loadings. All indexes reached the three criteria specified earlier.

**Table 1:** Means and standard deviations of 57 value items and of 19 values, standardized regression weights for the revised CFA model, reliabilities and Cronbach's alpha of the 19 factors.

**Value**	**Item**	**(1)**	**(2)**	**(3)**	**(4)**	**(5)**	**(6)**
		**Item mean**	**Item SD**	**Value mean**	**Value SD**	**Factor loading**	**Factor IoQ**
Self-direction-thought	SDT2	4.78	1.05	4.66	0.82	0.64	0.839
	SDT2	4.56	1.08			0.77	
	SDT3	4.63	0.98			0.58	
self-direction-action	SDA1	4.94	1.02	4.79	0.80	0.67	0.822
	SDA2	4.53	1.01			0.63	
	SDA3	4.90	1.05			0.62	
Stimulation	ST1	4.21	1.19	4.02	0.95	0.52	0.808
	ST2	3.53	1.26			0.55	
	ST3	4.31	1.27			0.78	
Hedonism	HE1^*^	3.90	1.25	4.57	1.00	—	0.801
	HE2	4.53	1.21			0.60	
	HE3	4.60	1.14			0.77	
Achievement	AC1	4.57	1.08	4.50	0.76	0.51	0.778
	AC2	4.39	1.11			0.71	
	AC3	4.43	1.07			0.72	
	AC4	4.61	0.96			0.56	
Power-dominance	POD1	3.43	1.21	3.57	0.98	0.51	0.677
	POD2^*^	3.19	1.36			—	
	POD3	3.72	1.24			0.59	
Power-resources	POR1^*^	3.14	1.36	3.40	1.07	—	0.707
	POR2	3.92	1.25			0.67	
	POR3	2.88	1.39			0.48	
Face	FAC1	4.75	1.11	4.54	1.02	0.66	0.638
	FAC2	4.61	0.96			0.79	
Security-personal	SEP1	5.16	1.00	5.10	0.82	0.61	0.771
	SEP2	5.04	0.94			0.68	
	SEP3^*^	4.22	1.19			—	
Security-societal	SES1	5.01	0.98	4.93	0.84	0.72	0.872
	SES2	4.89	1.06			0.78	
	SES3	4.89	1.01			0.66	
Tradition	TR1	4.11	1.19	4.17	0.91	0.45	0.812
	TR2	4.16	1.24			0.59	
	TR3	4.26	1.12			0.81	
Conformity-rules	COR1	4.69	1.04	4.66	0.83	0.64	0.820
	COR2	4.53	1.07			0.62	
	COR3	4.75	1.09			0.66	
Conformity-interpersonal	COI1	4.29	1.09	4.07	0.92	0.45	0.789
	COI2	3.84	1.18			0.69	
	COI3^*^	3.99	1.12			—	
Humility	HU1	4.23	1.17	4.39	0.90	0.41	0.717
	HU2	4.55	1.06			0.74	
	HU3^*^	4.02	1.20			—	
Universalism-nature	UNN1	4.72	1.02	4.68	0.86	0.78	0.900
	UNN2	4.59	0.99			0.74	
	UNN3	4.73	1.01			0.78	
Universalism-concern	UNC1	4.74	1.05	4.67	0.84	0.62	0.834
	UNC2	4.67	1.09			0.73	
	UNC3	4.60	1.07			0.62	
Universalism-tolerance	UNT1	4.81	1.01	4.74	0.81	0.66	0.852
	UNT2	4.76	0.99			0.75	
	UNT3	4.64	1.03			0.64	
Benevolence-caring	BEC1	4.98	0.99	4.99	0.71	0.46	0.795
	BEC2	5.29	0.89			0.74	
	BEC3	4.70	0.97			0.60	
Benevolence-dependability	BED1	4.60	1.01	4.85	0.78	0.40	0.677
	BED2	5.10	0.96			0.68	
	BED3^*^	4.49	1.06			—	

* Items dropped in CFA for which no standardized regression weight is therefore reported.

*IoQ = Index of Quality. IoQ (Saris and Gallhofer, 2007) refers to the correlation between the observed variables and the latent variable. The IoQ for each value was based on the retained items in the CFA and multidimensional scaling*.

We checked the correlations among the 19 value factors. Each pair correlated significantly, except for the correlation of power-resources with universalism–tolerance and with benevolence-caring. None of the correlations exceeded 0.80, showing that each of the 19 values could be distinguished by each of the latent factors. Column 5 of Table 1 provides the loading of each value item on its latent factors in the final revised model. Loadings of all items were significant (*p* < 0.001), with a value of at least 0.40. Sixteen of the 19 indexes of quality (IoQs) in Column 6 were >0.70, and three lower values were above 0.60. This demonstrated that the indices of all 19 values were reliable.

#### Behaviors

We performed the same procedure for the behavior items as in the value items. We allowed a reasonably good fit for at least two of the three criteria, which dropped 22 behavior items because of cross-loadings. Column 4 of Table 2 reports the IoQ reliabilities for each latent behavior factor, which showed the reliable indexes of 19 sets of behaviors. The bottom panel of Table 3 shows the fit statistics for four sets of revised behavior models. It was accepted that two CFIs seem slightly lower than the specified values and their RMSEA was better (Kenny and McCoach, 2003).

**Table 2:** Mean, standard deviations, and reliabilities of 19 behavior sets.

**Behavior set**	**(1)**	**(2)**	**(3)**	**(4)**
	**No. of items**	***M***	***SD***	**Factor IoQ**
Self-direction-thought	5(3)	2.27	0.65	0.806
Self-direction-Action	5(4)	1.61	0.66	0.812
Stimulation	4(3)	1.74	0.68	0.825
Hedonism	5(3)	2.03	0.63	0.794
Achievement	5(3)	2.00	0.61	0.781
Power-dominance	3	1.18	0.88	0.938
Power-resources	4	1.47	0.67	0.819
Face	5(3)	2.00	0.61	0.781
Security-personal	6(4)	2.37	0.62	0.787
Security-societal	4	2.68	0.67	0.819
Tradition	4(3)	2.00	0.72	0.849
Conformity-rules	5(3)	2.71	0.59	0.768
Conformity-interpersonal	4(2)	2.31	0.61	0.781
Humility	5(4)	2.22	0.56	0.748
Universalism-nature	4(3)	2.09	0.62	0.787
Universalism-concern	5(4)	2.26	0.6	0.775
Universalism-tolerance	4	2.41	0.61	0.781
Benevolence-caring	4(3)	2.63	0.53	0.728
Benevolence-dependability	4(3)	2.77	0.52	0.721

*In parentheses is the number of behavior items retained by the confirmatory factor analysis*.

**Table 3:** Confirmatory factor analyses: fit indexes for value and behavior sets.

**Model**	***χ***^**2**^	***df***	**CFI**	**RMSEA**	**SRMR**
**VALUES**					
1. Revised higher-order value model, openness: 4 factors 11 items, 4 factors	119.90	40.00	0.95	0.06	0.04
2. Revised higher-order value model, self-enhancement: 10 items, 4 factors	115.10	30.00	0.92	0.07	0.05
3. Revised higher-order value model, conservation: 15 items, 6 factors	233.00	84.00	0.92	0.06	0.05
4. Revised higher-order value model, self-transcendence: 14 items, 5 factors	223.10	73.00	0.94	0.06	0.04
**BEHAVIORS**					
1. Revised higher-order behavior model, openness: 13 items, 4 factors	206.20	60.00	0.90	0.07	0.06
2. Revised higher-order behavior model, self-enhancement: 13 items, 4 factors	131.40	58.00	0.95	0.05	0.04
3. Revised higher-order behavior model, conservation: 20 items, 6 factors	388.12	163.00	0.82	0.05	0.06
4. Revised higher-order behavior model, Self-transcendence: 17 items, 5 factors	290.60	112.00	0.88	0.06	0.05

*For all χ^2^ values, p < 0.01. Openness (self-direction-thought, self-direction-action, stimulation, and hedonism), self-enhancement (achievement, power-dominance, power-resources, and face), conservation (security-societal, security-personal, tradition, conformity-rules, conformity-interpersonal, and humility), and self-transcendence (universalism-nature, universalism-concern, universalism-tolerance, benevolence-caring, and benevolence-dependability)*.

### Circular Order of Values

We used the items retained in the CFA to perform the multidimensional scaling (MDS) analyses to test whether the values of minorities in Southwest China follow the theorized circle. All analyses were performed using SPSS 20.0 MDS Proxscal program (SPSS Inc., Chicago IL, USA), with ordinal proximity transformations, Euclidian distance measures, and *Z*-score transformations of factors. We based MDS analysis on a custom initial configuration of 19 equal points on the theorized circle to estimate the two-dimensional structures. The centered factor scores were used for the MDS analysis to control the individual differences in using response scales.

**Figure 1:**
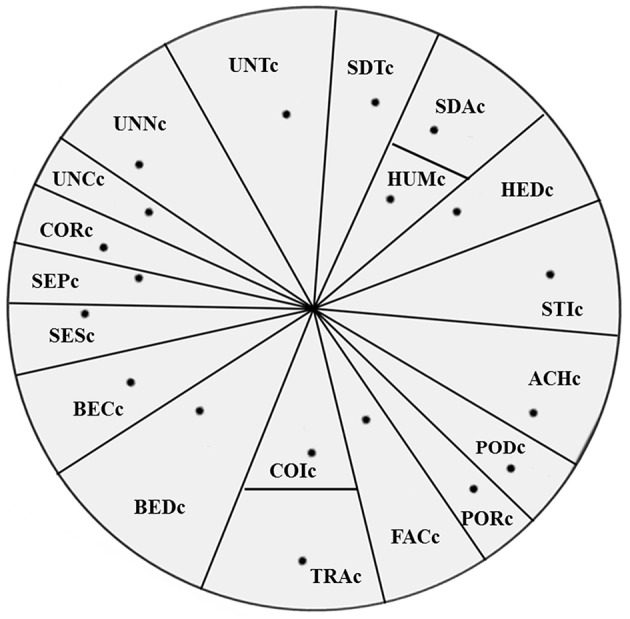
Multidimensional scaling plot of 19 value indexes based on the items retained in confirmatory factor analysis. Stress 1 = 0.221, dispersion accounted for = 0.95, Tucker's coefficient of congruence = 0.98.

As Figure 1 shows, the 19 values that are represented by labeled points are presented on a two-dimensional plot. The lines, regardless of whether they are straight or curved, form regions through continuous boundaries that do not intersect with other region boundaries with no substantive meaning. This projection was clearly split into 17 wedge-like regions. Of them, 12 value indexes accorded with the theorized motivational continuum. Hedonism switched locations with stimulation; humility and conformity-interpersonal were, respectively, inside the self-direction-action value region and the tradition value region; conformity-rules was adjacent to universalism region; and two benevolence facets not only switched locations with three universalism facets, but also with two security facets.

**Figure 2:**
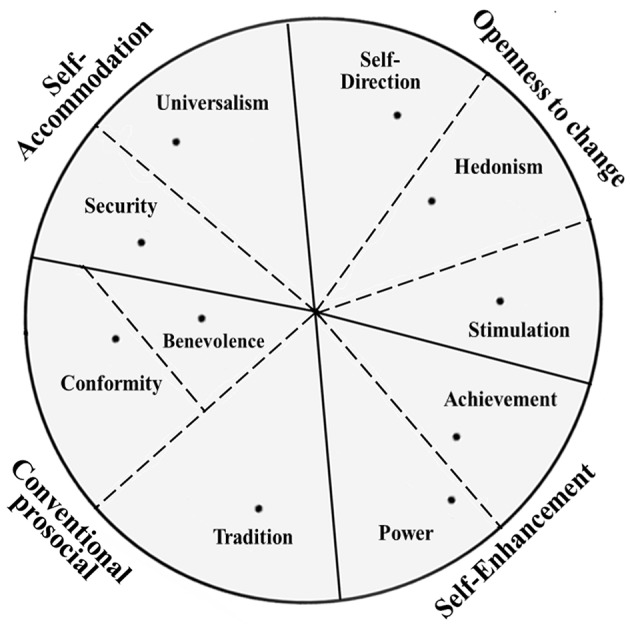
Multidimensional scaling plot of 10 recaptured values. Stress 1 = 0.171, dispersion accounted for = 0.97, Tucker's coefficient of congruence = 0.99.

We recaptured the 10 original values based on the observed orders. Conformity, security, power, self-direction, universalism, and benevolence were the six basic values that were split into more narrow values using the refined theory (Schwartz et al., 2012). We combined the narrow values into which it was originally split (e.g., the power-dominance and power-resources values were combined into the original power value). “Humility” and “face” values were merged, respectively, into self-direction and power. The result is presented in Figure 2. The projection is clearly interpretable. It reveals three deviation regions compared with the original theoretical circle. Benevolence and conformity were inside one sector, the latter toward the periphery; security replaced benevolence and was located between the universalism and conformity and benevolence; hedonism switched locations with stimulation; and tradition and conformity separately formed two distinct wedge-like regions.

### Associations Between Values and Behaviors

We ran 361 separate structural equation models to correlate 19 latent values with 19 latent behavior sets. The behavior items were those retained in the CFAs. For value items, we used the three original items to satisfy a minimum of three indexes for each latent variable. Table 4 shows results of the 361 relations.

**Table 4:** Correlations of 19 latent value factors with 19 latent behavior factors based on structural equation models.

	**_**b**_SDT**	**_**b**_SDA**	**_**b**_STI**	**_**b**_HED**	**_**b**_ACH**	**_**b**_POD**	**_**b**_POR**	**_**b**_FAC**	**_**b**_SEP**	**_**b**_SES**	**_**b**_TRA**	**_**b**_COR**	**_**b**_COI**	**_**b**_HUM**	**_**b**_UNN**	**_**b**_UNC**	**_**b**_UNT**	**_**b**_BEC**	**_**b**_BED**
SDT	0.39^***^	0.04	0.03	−0.13	0.48^***^	−0.15^*^	0.29	−0.21^**^	0.30^***^	0.40	0.17	0.51^***^	0.29^*^	0.07	0.33^***^	0.29^***^	0.38^***^	0.38^***^	0.48^***^
SDA	0.34^**^	**0.07**	0.01	0.00	0.46^***^	−0.21^***^	−0.33^***^	−0.31^***^	0.24^**^	0.38^***^	0.16^*^	0.48^***^	0.25^**^	0.03	0.25^**^	0.25^***^	0.41^***^	0.31^***^	0.45^***^
STI	0.54^***^	0.31^***^	0.66^***^	0.37^***^	0.40^***^	0.18^**^	0.18^*^	0.04	0.30^**^	0.22^**^	0.25^***^	0.18^**^	0.10^**^	0.06	0.35^***^	0.30^***^	0.44^***^	0.32^***^	0.22^**^
HED	0.30^**^	0.06	0.10	0.33^***^	0.24^**^	0.09	0.09	0.01	0.14^*^	0.19^**^	0.06	0.31^***^	0.21	0.08	0.01	0.03	0.23^**^	0.20^*^	0.21^**^
ACH	0.35^**^	0.14^*^	0.20^**^	0.08	0.79^***^	0.10	0.06	0.01	0.33^***^	0.49^***^	0.15^*^	0.37^***^	0.12^**^	−0.15^*^	0.24^**^	0.36^***^	0.29^***^	0.36^***^	0.44^***^
POD	0.033^***^	0.50^***^	0.47^***^	0.45^***^	0.46^**^	0.62^***^	0.52^***^	0.51^***^	0.34^**^	0.02	0.22^*^	0.07	0.20	0.07	0.10	0.23^***^	0.00	0.08	0.09
POR	0.20^*^	0.54^***^	0.40^***^	0.60^***^	0.31^**^	0.59^***^	0.74^***^	0.50^***^	0.18^**^	0.03	0.02	0.25	0.21	0.00	0.11	0.18^**^	0.04	0.02	0.18
FAC	0.18^*^	0.07	0.04	0.20^**^	0.33^***^	0.02	0.10	0.15*	0.12	0.37^***^	0.12	0.28^***^	0.30^**^	0.00	0.08	0.13^*^	0.22^**^	0.11	0.20^**^
SEP	0.16^*^	−0.18^*^	−0.21^**^	−0.18^*^	0.48^***^	−0.41^***^	−0.44^***^	−0.32^***^	0.29^**^	0.55^***^	0.16	0.52^***^	0.41^**^	0.09	0.21^*^	0.17^*^	0.36^***^	0.37^***^	0.51^***^
SES	0.19^**^	−0.22^***^	−0.27^***^	−0.21^**^	0.30^***^	−0.40^***^	−0.45^***^	−0.41^***^	0.16^*^	0.71^***^	0.47^***^	0.48^***^	0.33^***^	0.03	0.14^*^	0.18^**^	0.032^***^	0.33^***^	0.38^***^
TRA	0.29^***^	0.08	0.11	−0.00	0.32^**^	0.00	0.07	0.05	0.41^***^	0.37^***^	0.67^***^	0.17^*^	0.07	0.10	0.22^**^	0.30^***^	0.37^***^	0.21^**^	0.26^**^
COR	0.37^***^	−0.04	0.08	−0.22^**^	0.58^***^	−0.22^***^	−0.23^**^	−0.34^***^	0.54^***^	0.68^***^	0.42^***^	0.72^***^	0.44^***^	0.07	0.48^***^	0.42^***^	0.50^***^	0.54^***^	0.56^***^
COI	0.21^**^	0.01	0.05	0.13	0.50^***^	0.03	0.07	0.21^**^	0.21^**^	0.27^***^	0.12^*^	0.23^**^	0.80^***^	0.37^***^	0.07	0.14^*^	0.40^***^	0.38^***^	0.15^*^
HUM	0.41^***^	−0.12	0.06	0.04	0.33^**^	−0.21^**^	−0.22^*^	0.16	0.27^**^	0.42^***^	0.15	0.52^***^	0.53^***^	0.39^***^	0.31^**^	0.25^**^	0.60^***^	0.64^***^	0.41^**^
UNN	0.35^***^	−0.08	0.04	−0.13	0.47^***^	−0.16^**^	−0.23^***^	−0.27^***^	0.36*^**^	0.69^***^	0.50^***^	0.64^***^	0.32^***^	0.05	0.60^***^	0.47^***^	0.45^***^	0.50	0.38^***^
UNC	0.30^**^	−0.19^**^	−0.15^*^	−0.12	0.42^***^	−0.30^***^	−0.32^***^	−0.27^***^	0.25^**^	0.67^***^	0.19^*^	0.53^***^	0.42^***^	0.13	0.31^***^	0.25^***^	0.55^***^	0.47^***^	0.37^***^
UNT	0.46^***^	−0.07	0.01	−0.03	0.51^***^	−0.29^***^	−0.27^***^	−0.26^***^	0.31^***^	0.44^***^	0.30^**^	0.60^***^	0.45^***^	0.07	0.28^***^	0.33^***^	0.75^***^	0.53^***^	0.40^***^
BEC	0.15	−0.25^***^	−0.23^***^	−0.27^***^	0.30^**^	−0.36^***^	−0.45^***^	−0.31^***^	0.24^**^	0.48^***^	0.30^**^	0.43^***^	0.42^***^	0.04	0.33^***^	0.15^*^	0.40^***^	0.49^***^	0.62^***^
BED	0.22^*^	−0.23^*^	−0.19^*^	−0.10	0.40^**^	0.03	−0.47^**^	0.28	0.26^*^	0.42^***^	0.37^**^	0.47^***^	0.44^***^	0.11	0.11	0.21^*^	0.34^**^	0.49^***^	0.64^***^

* p < 0.05;

** p < 0.01;

*** p < 0.001.

*The prefix ‘b' denotes behavior. The bold values on the diagonal axis were the correlations of 19 latent values with their corresponding behavior sets. SDT, self-direction-thought; SDA, self-direction-action; STI, stimulation; HE, hedonism; ACH, achievement; POD, power-dominance; POR, power-resources; FAC, face; SEP, security-personal; SES, security-societal; TRA, tradition; COR, conformity-rules; COI, conformity-interpersonal; HUM, humility; UNN, universalism-nature; UNC, universalism-concern; UNT, universalism-tolerance; BEC, benevolence-caring; BED, benevolence-dependability*.

All correlations on the diagonal axis were significant (except self-direction-action). Eleven of 19 values correlated most strongly with its postulated behaviors than with the other 18 sets of behaviors. For seven values (stimulation, conformity-rule, power-dominance, power-resources, security-societal, tradition, benevolence-dependability), the correlations with their postulated behaviors were the strongest, followed by the behavior promoted by one of its adjacent values. Security-personal and benevolence-caring value correlated most strongly with its adjacent behavior, followed by its postulated behavior.

We also checked the correlation of each value with the behaviors that were presumably motivated by the opposing higher-order values in Table 4: four of the five self-transcendence values negatively correlated more strongly with the three of the four self-enhancement behaviors and with three of the four openness-to-change behaviors; two of the six conservation values negatively correlated more strongly with the three openness-to-change behaviors; face and two power values had the strongest negative correlation with the behaviors of three higher-order types beyond self-enhancement, including up to 29 negative correlations among 45 correlations (*p* < 0.05). Humility and stimulation value, which deviated from the theorized values orders, did not present negative correlations with behaviors motivated by their opposing higher-order values (*r*_s_ ≤ 0.08).

### Moderators of the Strength of the Value-Behavior Relations

#### Normative Pressure

According to Bardi and Schwartz (2003), the collective consensus about importance of value and desired behaviors was regarded as a potential source of normative pressure. They treated the average value importance and the average behavior frequency as the indicators of normative pressure. Hence, to test the moderating role of normative pressure on value-behavior relations, they used Spearman correlations to relate the ordering of 19 value-behavior relations with the ordering of 19 values importance and 19 behaviors frequency. This moderating role is validated if there existed significant negative correlations between the two pairs of correlations. A similar test was conducted in Schwartz and Butenko (2014), and was followed in the present study. However, as shown in Table 5, our results did not reveal significant correlations between them.

**Table 5:** Spearman rank correlations of the strength of value-behavior relations with value importance and behavior frequency.

	**Value-behavior correlations**		**Value importance**		**Behavior frequency**	
	***M***	**Rank**	***M***	**Rank**	***M***	**Rank**
Self-direction-thought	0.388	4	4.656	9	2.209	10
Self-direction-Action	0.066	19	4.787	5	1.700	16
Stimulation	0.663	8	4.017	17	1.680	17
Hedonism	0.328	15	4.569	11	1.731	15
Achievement	0.792	2	4.501	13	2.549	5
Power-dominance	0.623	10	3.574	18	1.176	19
Power-resources	0.742	4	3.400	19	1.468	18
Face	0.150	18	4.541	12	2.027	14
Security-personal	0.292	16	5.100	1	2.294	9
Security-societal	0.714	6	4.930	3	2.685	3
Tradition	0.668	7	4.174	15	2.355	7
Conformity-rules	0.717	5	4.657	10	2.970	1
Conformity-interpersonal	0.804	1	4.065	16	2.306	8
Humility	0.393	13	4.390	14	2.152	12
Universalism-nature	0.598	11	4.680	7	2.186	11
Universalism-concern	0.246	17	4.667	8	2.133	13
Universalism-tolerance	0.752	3	4.738	6	2.415	6
Benevolence-caring	0.490	12	4.989	2	2.551	4
Benevolence-dependability	0.640	9	4.852	4	2.966	2

#### Gender

To validate the moderating role of gender in value-behavior relations, we used the mean factor scores of values and behaviors, standardized gender, and formed the interaction terms between values and gender. We regressed each of the 19 behaviors on its corresponding value, gender, and the interaction term.

Seventeen of 19 values predicted their corresponding behavior significantly, as shown in Column 1 of Table 6. Gender predicted six behaviors significantly, as seen in Column 2: women reported behaving more frequently than did men in face, security-personal, and conformity-rules behaviors, and men reported behaving more frequently than women did in self-direction-action, conformity-interpersonal, and stimulation behaviors. The interaction items were significant for four sets of behaviors shown in Column 3, two self-direction domains, hedonism, and personal security, and all were stronger among women than men.

**Table 6:** Regressions of behavior factor scores on value factor scores, gender, and the Value × Gender interaction.

	**(1)**	**(2)**	**(3)**		**(4)**
	**Value beta**	**Gender beta (*p*)**	**Interaction**		**Adjusted *R^**2**^***
			**beta (*p*)**	**Confidence interval (95%)**	
Self-direction-thought	0.217^***^	0.006 (0.887)	0.647 (0.009)	(0.05, 0.33)	0.054
Self-direction-Action	0.028	−0.122 (0.005)	0.545 (0.044)	(0.01, 0.29)	0.017
Stimulation	0.454^***^	−0.078 (0.044)	0.227 (0.199)		0.208
Hedonism	0.144^***^	−0.042 (0.333)	0.566 (0.008)	(0.05, 0.29)	0.029
Achievement	0.450^***^	−0.008 (0.846)	0.249 (0.294)		0.200
Power-dominance	0.356^***^	−0.059 (0.149)	0.191 (0.215)		0.134
Power-resources	0.411^***^	−0.042 (0.298)	−0.020 (0.885)		0.171
Face	0.071	0.086 (0.049)	0.331 (0.108)		0.013
Security-personal	0.153^***^	0.160 (0.000)	0.644 (0.018)	(0.03, 0.33)	0.053
Security-societal	0.478^***^	−0.033 (0.382)	0.394 (0.087)		0.229
Tradition	0.372^***^	0.035 (0.382)	−0.346 (0.075)		0.139
Conformity-rules	0.464^***^	0.089 (0.020)	0.118 (0.596)		0.220
Conformity-interpersonal	0.335^***^	−0.096 (0.019)	−0.104 (0.591)		0.114
Humility	0.168^***^	−0.0 2 (0.563)	0.167 (0.452)		0.024
Universalism-nature	0.381^***^	−0.040 (0.318)	0.308 (0.172)		0.146
Universalism-concern	0.195^***^	−0.066 (0.123)	−0.159 (0.519)		0.036
Universalism-tolerance	0.425^***^	0.030 (0.441)	0.125 (0.599)		0.178
Benevolence-caring	0.270^***^	0.001 (0.982)	0.373 (0.219)		0.070
Benevolence-dependability	0.330^***^	−0.001 (0.986)	0.026 (0.922)		0.104

*** *p < 0.001*.

## Discussion

### Testing the Order of 19 Motivational Values

Each of the 19 values of minorities in Southwest China formed a single factor on the circular motivational continuum, which provided cross-cultural evidence on predictive and discriminant validity of the theory of refined value. However, our observed orders did not exactly correspond with the theorized orders (Schwartz et al., 2012), but were in line with our hypothesis. Schwartz (1992) excluded four of five Chinese samples from the analyses to achieve the ideal value order in 20 countries because they were poorly matched. To further understand the cultural root of the deviations from the theorized value orders, we discuss them in view of the Chinese culture.

Two conformity facets were separated in the circle. Interpersonal conformity was located at the same angle with tradition value and the former is inside the latter. This was consistent with the circular structure with the 10 original values, where conformity value was not split into two more narrowly defined values (Schwartz, 1992). China has always been regarded as a country with a highly collectivist culture (e.g., Hofstede, 1980; Tsai et al., 2006), in which conformity is essential. Previous studies have reported that conformity is a typical characteristic of Chinese traditional values (e.g., Yang, 1994). Additionally, “Harmony is prized” is a core value in the Chinese culture, in which interpersonal conformity plays an indispensable role (Zhai, 2004). Face and conformity are helpful to enhance interpersonal interactions and achieve interpersonal harmony. Consequently, the value projections of interpersonal conformity, tradition, and face demonstrated their shared motivational goal of harmony in the present study, which illustrated that Southwest minorities were deeply influenced by Chinese traditional culture (Li and Wu, 2017).

Schwartz et al. (2012) labeled the newly refined value “conformity-rules” as “conformity to laws, rules, and authority.” The traditional understanding of “rule of law” by Southwest minorities referred more to the obedience of the “laws of nature,” and their customs about nature worship also continue to the present (Zhang, 2017). Protecting nature means defending the safety of human existence. Thus, minorities conform not only to the laws and clan regulations, but also to the “law of nature.” The shared motivational emphasis promotes the conformity-rules value closer to universalism and security value.

Hedonism value prefers to pleasure or sensuous gratification and stimulation value prefers to excitement or change, and they differ in the degree of novelty (Schwartz, 1992). The reverse locations of hedonism and stimulation precisely reflect that minorities gradually become open to sensuous gratification and seek excitement, which is especially obvious among the youth of minorities (Zhou, 2009).

The “Five Constants” are the core virtues in the Chinese traditional value system, among which “benevolence” ranks first. It emphasizes that an individual should be humane to others, that is, “*Ren* (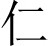, benevolence) means to be a man (*ren* 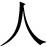)^1^” (Confucius/Legge, 2004, p. 10). It requires people to consider interests of others, and even give up “Small Ego” to achieve “Big Ego” in some special instances. Such virtues are consistent with the content of the items retained for benevolence value in PVQ, caring actively for the welfare of others. They reflect both benevolence and interpersonal conformity. The influences of Chinese traditional values on Southwest minorities also suggest cultural stability, which contributes to social security. The two observed benevolence values were located between traditions and conformity-interpersonal and security-societal in our motivational circle of values, which exactly revealed their shared motivational goals.

Humility value appears inside an unexpected polar angle, together with self-direction-action value. The result accorded with some reports that humility is undergoing a transformation from traditional self-insignificance to that with a modern sense of not renouncing self-interest based on mutual and equal benefit (e.g., Vera and Rodriguez-Lopez, 2004; Li, 2011), which is consistent with the developing orientation for minorities in Southwest China now. Moreover, humility also appears in a region different from its theorized projection (Schwartz et al., 2012). These suggestive findings await replication in future studies.

### Discriminating the 10 Original Values and Four Higher-Order Values

Compared with the theorizing circular projection of 10 values in Schwartz (1992), our MDS plot yielded eight discriminating regions plus a joint region of adjacent values as shown in Figure 2. We readjusted the grouping of the 10 values to constitute four higher-order types, which differed slightly from the theorized model. We grouped self-direction, hedonism, and stimulation to higher-order openness to change types; we grouped achievement and power to higher-order self-enhancement types; we grouped tradition, benevolence, and conformity into a new higher-order type that was named “conventional prosocial” (Schwartz and Boehnke, 2004); and security and universalism were grouped into another newly named higher-order type—self-accommodation. The first two higher-order types are identical to the theorized grouping. However, our observed orders did not exactly correspond with the theorized orders (Schwartz et al., 2012), which was in line with our hypothesis. The other newly named regroupings revealed the characterized higher-order value types of minorities in Southwest China, and accorded with the original intention of the refined value theory that researchers can regroup the four higher-order types to get optimal alternative combinations for research purposes.

### Associations Between Values and Behaviors

As shown in Table 4, the expected positive or strongest correlations happened between most of the 19 values and their a priori behavior, which validated the cross-cultural validity of stronger value-behavior relations. Additionally, the expected negative correlations of higher-order values with their postulated conflicting behaviors only emerged in 27.3% of the cases. The non-significant value-behavior correlation and the negative correlation deviations may be due to the following reasons.

First, some people practically rated values and behaviors relatively high or low regardless of content, and we did not standardize each participant's responses. This might bias the observed intercorrelations. Second, some studies reported that Chinese individuals prefer dialectical or compromising approaches, or find a “middle way” to deal with seemingly conflicting results (e.g., Peng and Nisbett, 1999), which is the “Golden Mean.” It implies that Chinese individuals tend to not show polarization in performing behaviors that conflict with their own asserted values. This may influence the negative relations of values with their postulated conflicting behaviors.

Finally, the items in EBQ were not derived from the context of minorities in Southwest China, and the opportunities for them to perform these behaviors in their daily lives were not universal compared to those of Russians (Schwartz and Butenko, 2014). For example, the items in self-direction–action behavior domain, in which the value-behavior relation was not found to be significant through two procedures. The behaviors of “Ignore a good idea because I wanted to choose what to do myself,” “Choose to do a task alone rather than with other people,” and “Do something my way even if someone might disapprove” were not consistent with common Chinese views, such as the Chinese proverbs of “Draw on collective wisdom and absorb all useful ideas” or “Hear all parties.” Those item contents are considered more as stubborn actions than as self-direction-action by the Chinese. In addition, two of five face items were deleted because of cross-loading after CFA. The retained items were “Feel offended when someone questioned my competence,” “Wonder whether people were gossiping about me,” “Feel anxious that someone might think I did something immoral.” These behaviors were less consistent with the meaning of face (Mianzi) that Chinese people believe understand as being respected, affirmed, flattered, or praised by others in their interpersonal relationships. This was also applicable to three other pairs of low value-behavior correlation (security-personal, hedonism, and universalism-concern). Hanel et al. (2018) reported that the typical value-expressive behaviors are different across nations despite the shared similar ideas on the abstract meaning of values and their importance, which may influence the relations of values and behaviors.

EBQ was the behavior set that corresponded to the 19 refined values. The joint application of PVQ and EBQ in this study could be a reference point to explore the relation of value to behavior in Southwest minorities, which would lay the foundation for us to explore more daily behaviors of the ethnic minority in Southwest China. Certainly, the resultant deviations might be also caused by translation variations, random error, and so on.

### Moderators of the Strength of the Value-Behavior Relations

#### Normative Pressure

Our result did not confirm the assumption that value-behavior relations were weaker under stronger normative pressure, similar to Schwartz and Butenko (2014). As reported in the previous research on the Chinese cultural feature of high collectivism identified by academia based on the Chinese tendency to value conformity, normative pressure would have a strong impact on the value-behavior relation. Actually, the definition of collectivism has always been controversial in academia (Rhee et al., 1996). Contemporary Chinese values are changing to have a more personal orientation (Zhou, 2009). Moreover, Chinese have their own unique concept of “individual-collective” and a psychological mechanism of integrating and coordinating the two poles. They can be one of them or be back to the middle again (Yang, 1997). Is China really “collectivistic” (Chen, 2017)? For minorities in Southwest China, individual values that have been deeply influenced by Confucianism are changing with social modernization (Ren, 2012; Li and Wu, 2017). Such values as self-direction, security, or an enterprising spirit tend to exist among ethnic minority teenagers (e.g., Zhang and Yang, 2003), and these rank among the top five in terms of value importance as seen in Table 5. The current findings reinforced the past research results, and demonstrated the reliability and predictive validity of the refined value theory.

#### Gender

In this study, the moderating effect of gender on value-behavior relations did not emerge in the benevolence domain for women and in the power domain for men, but in four unexpected domains: personal security, hedonism, and two self-direction domains. Interestingly, the value-behavior relationship was stronger among women than men in all these domains.

Evolutionary theory states that women had a great need to protect themselves and their infants during early childrearing. In most societies, compared to men, women are smaller, of lower status, and dependent greatly on others' support, which also make them more vulnerable than men (Schwartz and Rubel, 2005). Therefore, asserting the need for personal security is a more gender-appropriate and value-based instinct for women than for men, even without much cognitive processing.

Women in this study exhibited a stronger value-behavior relationship than did men in the hedonism domain, contrary to the conclusion in Schwartz and Rubel (2005). Evolutionary theory suggests that women must often forgo immediate gratification from many pleasurable sexual liaisons in responding to the needs of small children or avoiding relevant risks (Buss, 2003). This may lead women to have to suppress or reject sensual gratification. Later, in diverse historical and cultural backgrounds, sensual gratification was more seriously discouraged for women but accepted or even encouraged for men (Baumeister and Twenge, 2002). Such gender-role expectations push women away from hedonistic choices to avoid normative pressure unless these stressful choices are self-consciously motivated by their own internal values. Additionally, the description of hedonism in PVQ is precisely the pursuit of sensual pleasure.

The value-behavior relationship was stronger among women than men in self-direction value domains, which coincided with a popular “rake ear culture” in ethnic districts of Southwest China. “Rake (‘Pa' in Chinese) ear,” a unique dialect in China Sichuan, is used to describe a man who is suggestible, or more obedient to his wife, or “henpecked.” This shows that women are more involved in decision-making and executive than men in the family, which implies their stronger relation of self-direction value to behaviors. Our results may be just by chance but they embody the regional cultural characteristic in Southwest China.

Gender difference in power and benevolence domains did not appear in our study. Li and Chen (2003) suggested that the Chinese traditional culture based on “benevolence” may have been part of the basic personality structure of Chinese people, including men and women. Moreover, with the development of Chinese civilization, Chinese harmonious thought has continued to this day (Wu, 2014); harmony is motivationally opposed to the power value defined as control of people, materials, or social resources (Schwartz et al., 2012); this does not refer to men or women, but to the entire nation of China. More interesting is that our research results may provide befitting evidence for the above tests. The value-behavior associations suggested that two benevolence and power value factors correlated most strongly with its postulated or adjacent behaviors, and our participants rated security and benevolence as the most important value, and power as the least important value.

### Limitations and Future Research

There are some limitations that need to be noted. First, we only examined self-reported retrospective behaviors. Such reports probably attenuated some of the presented value-behavior associations due to incomplete memory and such motivated biases as consistency seeking or social desirability. However, self-reporters can base themselves on the full range of their experiences but not the shared experiences with the target or based on hearsay. Specific behavior frequency of self-ratings during the past year has also been shown to be quite accurate indicators (Gosling et al., 1998). Second, about the moderation mechanism of normative pressure in this study, we could not control the influences of some factors, such as age, education, and occupation, on normative pressure because of limitations in the method, which will be a direction for a future study. Third, we applied the non-native questionnaires set to minorities in Southwest China. This did not mean imposing original theorizing constructs on our respondents, but rather focusing on cross-cultural generality and differences. Our analyses support that values correlated strongly with behaviors chosen a priori are likely to express them. We will assess the reliability and content validity reported in this study, of the strongest correlations of value with its postulated behaviors in future research on Southwest minority in China. Based on the findings that diverged from the theory, we compiled a set of EBQ grounded in the Chinese context to match the motivational circle of the 19 refined values. Uniqueness that distinguishes each minority makes it more complicated to extract universality of behavior frequency among diverse minorities. Future researchers could focus on the common behaviors that apply across minorities or a continuous study of a single distinctive minority. It would be more meaningful and attractive to test relations between the refined values and sets of native homogenous behaviors within diverse minorities.

## Conclusion

With the aim of exploring psychological features of Chinese minorities against diverse cultural context, we examined the relations of values to behaviors among minorities in Southwest China by confirming the predictive and discriminant validity of the refined value theory. Despite some limitations, this study is a pioneering step in understanding the relationship between the values and behavior of Chinese minorities. The strongest relations of 11 of 19 values to their postulated behaviors and the consistent value-ordering with the theorized circle provide cross-cultural evidence to the universality of the refined value theory; however, such seemingly contradictory values as conformity, humility, self-direction, stimulation, benevolence, and tradition motivated more harmonious behaviors in Chinese minorities, in which perhaps the “Golden Mean” asserted by Confucianism rather than the normative pressure plays a role. Our results did not explain the moderating role of normative pressure on value-behavior relations. The moderating role of gender in the value-behavior relations also reflected the regional cultural uniqueness of minorities in Southwest China. The result from this study contribute to explaining ethnic behaviors corresponding to value, and provide psychological evidence to understand the uniqueness of Southwest minorities.

## Attachment

There are 56 ethnicities in China, including 55 officially defined ethnic minority groups and one majority group, ethnic Han. These ethnic minorities are so named because the population of each minority is much smaller than that of the Han ethnicity, which is the most populous ethnic group of China and represents the Chinese universal and mainstream culture. For instance, according to the Chinese sixth population census data (Chinese National Bureau of Statistics, 2010), there are more than 1 billion 200 million ethnic Han, more than 9 million 400 thousand ethnic Miao, but only 7,000 Durong (one of the less populous minority groups in China and the least populous minority in Yunnan).The minority groups either inhabited compactly in a little community or mixed sporadically and dispersedly with other ethnicities in a large region, namely, “large diaspora, small settlement, and interlaced inhabitation.” For instance, Miao people scatter across China, 42.1% of them inhabit compactly in Guizhou, and interlace sporadically and dispersedly with other ethnicities. This feature is not observed among ethnic Han. Despite such distributions of Chinese minorities, almost every minority group has its own ethnic uniqueness from others, such as historical origin, ethnic customs, language, and food and clothing, which may give birth to something that they value and carry forward. For instance, Durong has its own traditional festival, called “Kaque wow” in Durong language; and ethnic Naxi is also known for its music, frescoes, and wedding and food customs. These are three different features of minority groups and ethnic Han in China, which are also applicable to minority groups in Southwest China.

## Ethics Statement

The study was carried out in accordance with the recommendations of the Ethics Committee of Southwest University with written informed consent from all participants before the surveys. All participants gave written informed consent in accordance with the Declaration of Helsinki. The protocol was approved by the Ethics Committee of Southwest University.

## Author Contributions

YY and YZ conceived and designed the study. YY collected and analyzed the data, and drafted the initial manuscript. JZ contributed to the data analyses. PF reviewed the manuscript. GJ and YL participated in issuing and collecting the questionnaires. YZ provided the critical comments and revision. All authors approved the final version of the manuscript for submission.

### Conflict of Interest Statement

The authors declare that the research was conducted in the absence of any commercial or financial relationships that could be construed as a potential conflict of interest.

## Footnotes

^1^The two words are etymologically related homonyms.
